# Supplementary material for: Histopathological Gastric Cancer Detection on GasHisSDB Dataset Using Deep Ensemble Learning
Source: Diagnostics (Basel). 2023 May 18;13(10):1793. doi: 10.3390/diagnostics13101793 (PMC10217020; doi:10.3390/diagnostics13101793)
Supplement: Supplementary file 1 [file diagnostics-13-01793-s001.zip › Minor Revision Clean - Diagnostic Journal Supplementary Materials.pdf]

SUPPLEMENTARY MATERIAL FOR THE ARTICLE

Histopathological Gastric Cancer Detection on GasHisSDB Dataset using Deep Ensemble Learning

Ming Ping Yong, B.S, [a], Yan Chai Hum, PhD, [a], Khin Wee Lai, PhD, [b], Ying Loong Lee, PhD, [a] Choon-Hian Goh, PhD, [a], Wun-She Yap, PhD, [a], Yee Kai Tee, DPhil, [a*].

[a] Lee Kong Chian Faculty of Engineering and Science, Universiti Tunku Abdul Rahman, Malaysia

[b] Department of Biomedical Engineering, Faculty of Engineering, University of Malaya, Jalan Universiti, Kuala Lumpur, Malaysia

[*]Corresponding Authors

Yee Kai Tee
Email address: teeyeekai@gmail.com

This Supplementary Material file contains:

I. Complete Model Validation Performance Tables on the GasHisSDB Gastric Dataset
II. Complete Test Performance Tables by the Best Performing Models of Our Study and the Previous State-of-the-art Studies on the GasHisSDB Gastric Dataset
III. Complete Model Test Performance Tables on the HICL Larynx Dataset

I. Complete Model Validation Performance Tables on the GasHisSDB Gastric Dataset

The pre-trained networks or base models for ensemble model building were selected based on their accuracy performance on the validation set. As shown in Table S1, for 80-pixels sub-database, EfficientNetB0, DenseNet169, and EfficientNetB1 were the top three models with the highest validation accuracies, followed by DenseNet121 and MobileNet.

Table S1. Models performance on 80-pixels sub-database validation set. The best-achieved results are bold. All metrics are measured in % unit.

Model	Accuracy	AUC	Precision	Recall	Specificity	F1-score
MobileNet	96.06	95.97	95.22	95.39	96.54	95.31
MobileNetV2	95.49	95.08	96.57	92.54	97.63	94.51
**EfficientNetB0**	**96.75**	**96.74**	**95.66**	**96.64**	**96.83**	**96.15**
EfficientNetB1	96.66	96.57	96.01	96.03	97.11	96.02
DenseNet121	96.65	96.35	97.49	94.45	98.25	95.95
DenseNet169	96.73	96.74	95.44	96.83	96.66	96.13
InceptionV3	94.75	94.72	93.04	94.56	94.88	93.79
Xception	95.80	95.71	94.86	95.15	96.27	95.00

For the 120-pixels sub-database, DenseNet169, DenseNet121, and EfficientNetB1 were the top three models with the highest validation accuracies, followed by EfficientNetB0 and MobileNetV2 (Table S2).

Table S2. Models performance on 120-pixels sub-database validation set. The best-achieved results are bold. All metrics are measured in % unit.

Model	Accuracy	AUC	Precision	Recall	Specificity	F1-score
MobileNet	97.20	97.03	96.66	96.21	97.84	96.43
MobileNetV2	97.51	97.58	95.87	97.89	97.26	96.87
EfficientNetB0	97.72	97.75	96.35	97.91	97.59	97.13
EfficientNetB1	97.82	97.77	96.94	97.55	98.00	97.24
DenseNet121	98.12	97.97	97.90	97.30	98.64	97.60
**DenseNet169**	**98.21**	**98.12**	**97.75**	**97.71**	**98.54**	**97.73**
InceptionV3	96.72	96.63	95.48	96.23	97.04	95.85
Xception	97.14	97.03	96.24	96.51	97.55	96.38

As for the 160-pixels sub-database, the top three models were DenseNet121, DenseNet169, and EfficientNetB1, followed by EfficientNetB0 and MobileNetV2 as the top five models as shown in Table S3.

Table S3. Models performance on 160-pixels sub-database validation set. The best-achieved results are bold. All metrics are measured in % unit.

Model	Accuracy	AUC	Precision	Recall	Specificity	F1-score
MobileNet	97.99	97.74	98.59	96.41	99.06	97.49
MobileNetV2	98.39	98.34	97.94	98.09	98.60	98.02
EfficientNetB0	98.48	98.45	98.02	98.24	98.65	98.13
EfficientNetB1	98.50	98.44	98.17	98.13	98.75	98.15
**DenseNet121**	**99.10**	**99.01**	**99.23**	**98.55**	**99.48**	**98.89**
DenseNet169	98.93	98.77	99.42	97.94	99.61	98.67
InceptionV3	98.24	98.20	97.68	97.98	98.41	97.83
Xception	97.79	97.70	97.32	97.21	98.18	97.27

The top three and five models mentioned above were selected as the base models for the ensemble models in the respective sub-database for testing.

II. Complete Test Performance Tables by the Best Performing Models of Our Study and the Previous State-of-the-art Studies on the GasHisSDB Gastric Dataset

As shown in Table S4 to

Table **S6**, the proposed ensemble models consistently outperformed the previous state-of-the-art studies in various metrics including accuracy, precision, recall, specificity, and F1-score, except the AUC on the 160-pixels sub-database.

Table S4. Comparison between the best performing models from our study and the previous state-of-the-art studies on 80-pixels sub-database. The best-achieved results are bold. All metrics are measured in % unit.

Model	Accuracy	AUC	Precision	Recall	Specificity	F1-score
VGG16 [2]	96.12	-	94.2	96.3	96.0	95.2
ResNet50 [2]	96.09	-	96.2	94.0	97.5	95.1
MCLNet [3]	96.28	-	94.5	96.4	96.2	95.4
**Ensemble-UA5**	**97.72**	**97.65**	97.39	**97.18**	98.12	**97.28**
**Ensemble-WA5**	97.69	97.59	**97.54**	96.95	**98.23**	97.24

Table S5. Comparison between the best performing models from our study and the previous state-of-the-art studies on 120-pixels sub-database. The best-achieved results are bold. All metrics are measured in % unit.

Model	Accuracy	AUC	Precision	Recall	Specificity	F1-score
VGG16 [2]	96.47	-	96.7	94.0	98.0	95.3
ResNet50 [2]	95.94	-	96.2	93.0	97.8	94.6
MCLNet [3]	97.95	-	97.7	96.9	98.6	97.3
**Ensemble-UA5**	98.68	**98.61**	98.38	**98.27**	98.95	98.32
**Ensemble-WA5**	**98.69**	98.59	**98.54**	98.13	**99.06**	**98.33**

Table S6. Comparison between the best performing models from our study and the previous state-of-the-art studies on 160-pixels sub-database. The best-achieved results are bold. All metrics are measured in % unit.

Model	Accuracy	AUC	Precision	Recall	Specificity	F1-score
VGG16 [2]	95.90	-	93.80	96.00	95.90	94.90
ResNet50 [2]	96.09	-	94.60	95.60	96.40	95.10
**InceptionV3** [1]	98.83 ± 0.05	**99.90 ± 0.01**	-	-	-	-
InceptionV3 + ResNet50(Ensemble model - feature concatenation) [1]	98.80 ± 0.12	99.89 ± 0.03	-	-	-	-
Local-global feature fuse network [4]	96.81	**-**	97.18	94.66	98.21	95.91
MCLNet [3]	97.85	-	96.80	97.80	97.90	97.30
**Ensemble-UA5**	**99.20**	99.14	**99.23**	**98.80**	**99.48**	**99.01**
Ensemble-WA5	99.16	99.09	99.19	98.72	99.45	98.96

III. Complete Model Test Performance Tables on the HICL Larynx Dataset

To prove the effectiveness and robustness of our proposed ensemble models and also to show the proposed work is not sample/dataset limited, we further experimented the proposed models on the Histology Image Collection Library (HICL) histopathology larynx dataset. This is a multi-class dataset consists of Grade I, II and III tumors, and has a total of 224 images across all three classes. The dataset summary and samples are illustrated in Figure S1.

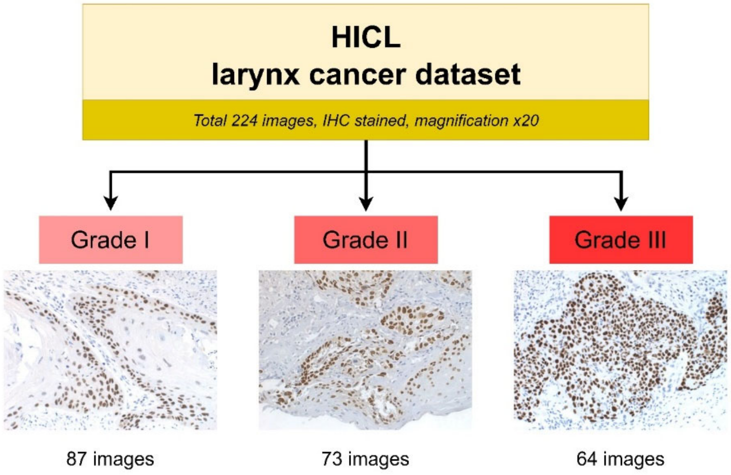

Figure S1. HICL dataset samples and summary.

For data preprocessing, the similar data augmentation method as described in Section 3.2.1 was applied to expand the dataset size to eight times of the original dataset size since the original dataset size was small. The empty patch removal was also performed using similar setting as in Section 3.2.1. The pre-processing steps were done because these had been found useful to increase the model performance. The same models and experiment setting as in Section 3.2 were used too but there was a slight modification to the output softmax layers of the models; they were set to 3 nodes instead of 2, to cater for the 3 classes in the extended experiment dataset. The dataset distribution is shown in Table S7.

Table S7. HICL dataset distribution after data pre-processing (empty patch removal and data augmentation).

Dataset	Number of patches			
	Training set	Augmented training set	Validation set	Testing set
534 x 400 pixels	581	4,648	145	170
1067 x 800 pixels	2,809	22,472	716	896

As shown in Table S8, the ensemble model Ensemble-MV5 achieved the highest accuracy of 96.47% on the 534 x 400 pixels dataset. For other metrics, the Ensemble-MV5 achieved 97.29% AUC, 96.56% precision, 96.39% recall, 98.20% specificity, and 96.44% F1-score.

Table S8. Performance of the different deep learning models performance on the testing set of 534 x 400 pixels dataset. The first result is the state-of-the-art result, the rest are the tested models in this study. The best-achieved results are bold. For the ensemble learning models, WA stands for weighted averaging, UA stands for unweighted averaging and MV stands for majority voting; and the 3 and 5 at the end of the ensemble models refer to top 3 or 5 base models. All metrics are measured in % unit.

Model	Single class accuracy			Accuracy	AUC	Precision	Recall	Specificity	F1-score
	G1	G2	G3						

LPCANet [5]	81.18	74.46	60.42	73.18	88.26	74.04	73.18	-	72.90
MobileNet	77.27	85.45	73.47	78.82	84.05	78.54	78.73	89.37	78.58
MobileNetV2	89.39	83.64	89.80	87.65	90.70	87.53	87.61	93.78	87.56
EfficientNetB0	89.39	94.55	95.92	92.94	94.89	92.88	93.29	96.49	93.01
EfficientNetB1	87.88	92.73	89.80	90.00	92.58	89.93	90.13	95.02	89.96
DenseNet121	93.94	81.82	93.88	90.00	92.51	89.95	89.88	95.14	89.61
**DenseNet169**	96.97	83.64	83.67	88.82	91.08	90.73	88.09	94.06	88.91
InceptionV3	86.36	89.09	77.55	84.71	88.27	85.29	84.34	92.21	84.57
Xception	89.39	90.91	83.67	88.24	91.05	88.42	87.99	94.11	88.07
Ensemble-WA3	96.97	90.91	95.92	94.71	95.93	94.94	94.60	97.27	94.73
Ensemble-WA5	98.48	89.09	95.92	94.71	95.90	94.76	94.50	97.31	94.57
Ensemble-UA3	96.97	90.91	95.92	94.71	95.96	94.76	94.60	97.31	94.64
Ensemble-UA5	98.48	89.09	95.92	94.71	95.90	94.76	94.50	97.31	94.57
Ensemble-MV3	96.97	87.27	95.92	93.53	94.99	94.26	93.39	96.60	93.66
**Ensemble-MV5**	**98.48**	**92.73**	**97.96**	**96.47**	**97.29**	**96.56**	**96.39**	**98.20**	**96.44**

Meanwhile, for 1067 x 800 pixels dataset, the ensemble models, Ensemble-WA5 and Ensemble-UA5 achieved the same highest accuracy, 97.99%. They also had the same performance for other metrics such as 98.47% AUC, 98.01% precision, 97.96% recall, 98.98% specificity, and 97.99% F1-score as presented in Table S9.

Table S9. Performance of the different deep learning models performance on the testing set of 1067 x 800 pixels dataset. The first result is the state-of-the-art result, the rest are the tested models in this study. The best-achieved results are bold. For the ensemble learning models, WA stands for weighted averaging, UA stands for unweighted averaging and MV stands for majority voting; and the 3 and 5 at the end of the ensemble models refer to top 3 or 5 base models. All metrics are measured in % unit.

Model	Single class accuracy			Accuracy	AUC	Precision	Recall	Specificity	F1-score
	G1	G2	G3						
LPCANet [5]	81.30	89.40	78.50	83.15	94.87	83.50	83.10	-	83.10
MobileNet	92.82	89.00	96.89	92.75	94.63	92.78	92.90	96.35	92.76
MobileNetV2	93.10	96.91	94.55	94.75	96.11	94.72	94.85	97.37	94.77
EfficientNetB0	95.40	94.85	97.67	95.87	96.95	95.80	95.97	97.93	95.87
EfficientNetB1	94.83	94.50	95.72	94.98	96.26	94.83	95.02	97.51	94.92
DenseNet121	97.70	95.88	96.11	96.65	97.42	96.77	96.56	98.28	96.66
**DenseNet169**	96.84	94.16	97.28	96.09	97.05	96.13	96.09	98.02	96.09

InceptionV3	91.95	94.85	93.77	93.42	95.10	93.38	93.52	96.68	93.45
Xception	93.97	91.75	94.94	93.53	95.14	93.51	93.55	96.73	93.52
Ensemble-WA3	98.56	96.22	97.67	97.54	98.11	97.66	97.48	98.73	97.56
**Ensemble-WA5**	**98.28**	**97.94**	**97.67**	**97.99**	**98.47**	**98.01**	**97.96**	**98.98**	**97.99**
Ensemble-UA3	98.56	96.22	97.67	97.54	98.11	97.66	97.48	98.73	97.56
**Ensemble-UA5**	**98.28**	**97.94**	**97.67**	**97.99**	**98.47**	**98.01**	**97.96**	**98.98**	**97.99**
Ensemble-MV3	98.28	96.22	97.67	97.43	98.03	97.54	97.39	98.67	97.46
Ensemble-MV5	97.99	97.94	97.67	97.88	98.39	97.90	97.86	98.92	97.88

The proposed ensemble models easily beat the best reported results in the literature [5]. All these demonstrated the ability and generalization of our proposed ensemble models to handle different histopathology datasets of different organ origins, different staining methods and multi-class classification tasks.

References

1. Springenberg, M.; Frommholz, A.; Wenzel, M.; Weicken, E.; Ma, J.; Strodthoff, N. From CNNs to Vision Transformers -- A Comprehensive Evaluation of Deep Learning Models for Histopathology. **2022**, doi:2204.05044.
2. Hu, W.; Li, C.; Li, X.; Rahaman, M.M.; Ma, J.; Zhang, Y.; Chen, H.; Liu, W.; Sun, C.; Yao, Y.; et al. GasHisSDB: A New Gastric Histopathology Image Dataset for Computer Aided Diagnosis of Gastric Cancer. *Comput Biol Med* **2022**, *142*, 105207, doi:10.1016/J.COMPBIOMED.2021.105207.
3. Fu, X.; Liu, S.; Li, C.; Sun, J. MCLNet: An Multidimensional Convolutional Lightweight Network for Gastric Histopathology Image Classification. *Biomed Signal Process Control* **2023**, *80*, 104319, doi:10.1016/j.bspc.2022.104319.
4. Li, S.; Liu, W. LGFFN-GHI: A Local-Global Feature Fuse Network for Gastric Histopathological Image Classification. *Journal of Computer and Communications* **2022**, *10*, 91–106, doi:10.4236/jcc.2022.1011007.
5. Zhou, X.; Tang, C.; Huang, P.; Mercaldo, F.; Santone, A.; Shao, Y. LPCANet: Classification of Laryngeal Cancer Histopathological Images Using a CNN with Position Attention and Channel Attention Mechanisms. *Interdiscip Sci* **2021**, *13*, 666–682, doi:10.1007/s12539-021-00452-5.
